# First report of multi-drug resistant tuberculosis in a systemic lupus erythematosus patient

**DOI:** 10.1186/s13104-015-1302-x

**Published:** 2015-08-06

**Authors:** Kunchok Dorjee, Kerry L Dierberg, Tsetan D Sadutshang, Arthur L Reingold

**Affiliations:** Division of Epidemiology, School of Public Health, University of California, Berkeley, 113 Haviland Hall #7358, Berkeley, CA 94720-7358 USA; Johns Hopkins University School of Medicine, Baltimore, MD USA; The Tibetan Delek Hospital, Dharamshala, Himachal Pradesh India

**Keywords:** Tuberculosis, Multi-drug resistant tuberculosis, Systemic lupus erythematosus, Lupus

## Abstract

**Background:**

Treatment of a multi-drug resistant tuberculosis (MDR-TB) patient is clinically challenging, requiring a minimum of 18 months of therapy. Its occurrence in a systemic lupus erythromatosus (SLE) patient may complicate management of both MDR-TB and SLE. This is the first descriptive report of MDR-TB in an SLE patient.

**Case presentation:**

A 19-year old female receiving long-term prednisolone for SLE was diagnosed with MDR-TB. She was started on MDR-TB treatment regimen and prednisolone was replaced with azathioprine. After an initial response to therapy, patient experienced a flare of lupus symptoms. Imaging studies revealed avascular necrosis of right femoral head. She was then treated with intravenous methyl-prednisolone, followed by maintenance corticosteroid. Azathioprine was discontinued due to hematological toxicity and failure to control SLE. Her symptoms of lupus regressed and did not re-occur for the duration of her MDR-TB treatment. Patient was declared cured of MDR-TB after 18 months of ATT. She is currently scheduled for a total hip replacement surgery.

**Conclusions:**

This case highlights the challenges of simultaneously managing MDR-TB and SLE in a patient due to their over-lapping signs and symptoms, drug–drug interactions, and the need for use of immunomodulatory agents in the absence of standard guidelines and documented previous experiences. Our experience underscores the importance of appropriate selection of treatment regimens for both MDR-TB and SLE.

## Background

Systemic lupus erythematosus is a chronic autoimmune disease. TB incidence and mortality rate are high in SLE patients [1, 2]. With the surge of drug resistant TB globally, more SLE patients may develop MDR-TB [3]. Through this case report, we have shown a successful management of an MDR-TB case in an SLE patient, despite initial treatment challenges.

## Case presentation

**Fig. 1:**
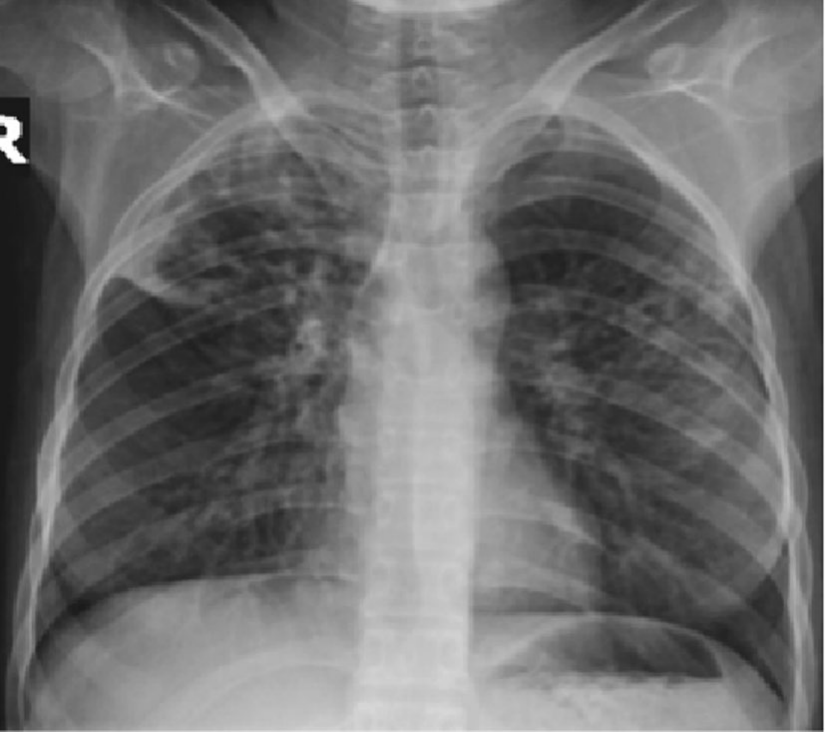
Chest radiograph showing bilateral upper and mid-lung infiltrates due to MDR-TB in a patient suffering from SLE.

In November 2010, a 19-year-old ethnically Tibetan female with SLE was referred to us with sputum culture and drug susceptibility test (DST) results, which demonstrated multi-drug resistant pulmonary TB with resistance to isoniazid, rifampicin, ethambutol, streptomycin, ethionamide and susceptibility to kanamycin, capreomycin, ofloxacin, moxifloxacin, pyrazinamide, para-aminosalicylic acid and clofazimine. While there was no clear history of contact with TB cases, she was residing in a boarding school where cases of MDR-TB have occurred over the past few years. Prior to receiving the DST result, she had taken category I anti-tubercular treatment (ATT) with isoniazid, rifampicin, pyrazinamide, and ethambutol for 45 days without response. She presented to us with fever, cough, and generalized weakness and was sputum smear positive for acid-fast bacilli. Chest radiograph demonstrated bilateral upper and mid-lung infiltrates with cavitary lesions (Fig. 1).

In 2006, she was diagnosed with SLE after presenting with malar rash, photosensitivity, and polyserositis (pleural effusion, pericardial effusion, ascites). Tests for anti-nuclear antibody and anti-double stranded DNA antibody were positive. Since then, she had been on methyl prednisolone and hydroxychloroquine (HCQ). For the year prior to presentation she was taking methyl prednisolone 8 mg on alternate days and HCQ 200 mg twice daily.

**Fig. 2:**
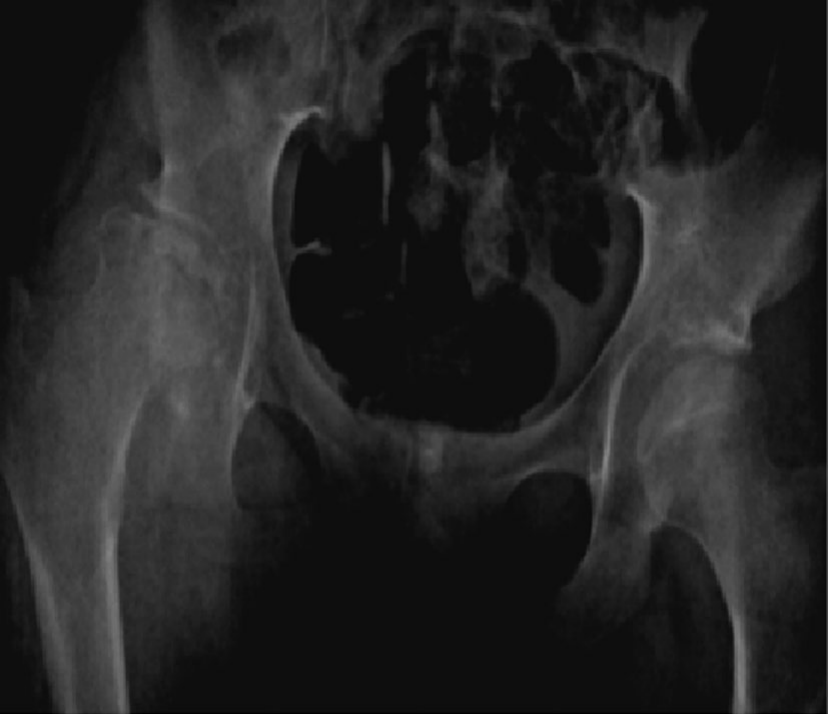
Radiograph showing elevated right hemipelvis and irregular contour of right hip-joint with lytic and sclerotic lesions in the femoral head in an SLE patient diagnosed with MDR-TB.

On November 24, 2010, we started MDR-TB treatment with kanamycin 600 mg, levofloxacin 750 mg, para-aminosalicylic acid 8 g, cycloserine 750 mg, pyrazinamide 1,250 mg, and clofazimine 100 mg. Her weight was 42 kg. A dose reduction of methyl prednisolone was initiated and azathioprine 50 mg was introduced, with a plan to gradually increase the dose to 100–125 mg. After gradual taper, methyl prednisolone was stopped after 30 days. Her symptoms initially improved with resolution of fever and subsidence of cough. Sputum-smear became negative. However, after 2 months of MDR-TB therapy, she complained of difficulty walking with right hip pain radiating to the knee, worse with movement, and had developed a limp. On examination, there was notable shortening of right lower extremity with muscle wasting in the thighs and legs. In a week’s time, she developed butterfly rash on her cheeks, alopecia, and increased difficulty in walking. Radiograph of the hip-joints showed lytic and sclerotic lesions of the right femoral head with irregular contour (Fig. 2). MRI of the hip-joints showed avascular necrosis of bilateral femoral heads, right greater than left, with minimal effusion of right hip-joint. Her hemoglobin had dropped from 12 to 7.9 gm% and platelet count had dropped from 260,000 to 68,000/µl. She was referred for multi-disciplinary consultation, which revealed no evidence of central nervous system involvement or lupus nephritis. Methyl prednisolone IV pulse therapy was started, followed by an oral prednisolone taper. Azathioprine was stopped in view of possible myelosuppression resulting in anemia and thrombocytopenia. The patient clinically improved, with resolution of the skin rash and arthralgias. She was put on maintenance prednisolone dose of 7.5 mg, HCQ 200 mg twice daily, calcium supplements and once-weekly alendronate with vitamin D_3_. MDR-TB treatment was continued. Two months after stopping Azathioprine, her hemoglobin level and platelet count had increased to 10.1 gm% and 139,000/µl respectively. During follow up orthopedic consultations, no progression of osteonecrosis of hip joint was observed, and total hip joint replacement was recommended after completion of MDR-TB treatment. Injection kanamycin was stopped after 9 months of ATT and pyrazinamide was stopped after 1 year of ATT. Consecutive sputum smears were negative. She had seven negative cultures for TB since the start of MDR-TB treatment. After 18 months of ATT, she was declared cured and TB treatment was stopped. She is currently being planned for a hip replacement surgery.

## Discussion

To our knowledge, this is the first reported case of MDR-TB in an SLE patient. Immunosuppression from chronic steroid therapy and living in a high TB and MDR-TB prevalence area were important risk factors for development of MDR-TB in our SLE patient. A high rate of MDR-TB has been previously reported in the Tibetan population living in India [4]. Diagnosis and management of TB and especially MDR-TB in an SLE patient pose several challenges. We think there are four major points to highlight: (1) Signs/symptoms of TB and SLE can mimic each other posing a diagnostic challenge, (2) Drug toxicities of MDR-TB treatment, including skin rash, arthralgias, nephrotoxicity, anemia, thrombocytopenia and CNS symptoms could also mimic signs/symptoms of SLE and thus complicate management, (3) Drug-drug interactions and overlapping toxicities can complicate management of SLE and MDR-TB, especially in the face of mycobacterial resistance to fluoroquinolones and/or aminoglycosides where more toxic drugs like linezolid may have to be used, which share similar hematological toxicity profile with immuno-suppressive agents such as azathioprine used for SLE [5], and (4) Absent clear-cut guidelines, use of immunosuppressant agents and their dose adjustments in an SLE patient with MDR-TB could be clinically challenging for the treating physician.

In this patient, the hip joint involvement could be either due to long-term steroid therapy, osteonecrosis from SLE or MDR-TB of hip-joint, though the revelation of avascular necroses of bilateral femoral heads on MRI, and development of leg/joint pains while receiving ATT suggested chronic steroid therapy as the etiology. It was difficult to clearly distinguish between chronic steroid therapy and SLE as the cause. We had some concern with the use of pyrazinamide given her hip-joint involvement and leg pain since pyrazinamide can lead to hyperuricemia and arthralgias [6]. However, since our patient’s symptoms subsided soon after re-introduction of corticosteroid, we decided to continue with pyrazinamide for a year, carefully weighing the risks and benefits. Our patient was fortunate in that she was susceptible to the core second-line anti-TB drugs: fluoroquinolones and aminoglycosides. The SLE flare she experienced during the course of MDR-TB treatment was likely due to the attempted withdrawal of glucocorticoid rather than worsening TB given her initial improvement after MDR-TB treatment, continued sputum-smear negativity, and the rapid response to restarting glucocorticoid. Through out the treatment course, we were vigilant of any signs and symptoms of neuropsychiatric lupus such as depression, since our patient was also receiving cycloserine for MDR-TB that can also cause depression and trigger suicidal tendencies [7]. In summary, given the documented poorer outcomes of MDR-TB treatment in other immuno-compromised states [8, 9], it becomes important that initiation and dose adjustment of corticosteroids and immuno-suppressants for SLE are cautiously done taking care to maintain appropriate response to ATT and also achieve a sustained remission of SLE. Additionally, due to overlapping drug toxicities, meticulous selection of MDR-TB treatment regimen in an SLE patient is important.

## Conclusion

Although SLE and MDR-TB may be a rare combination, rise of global incidence of MDR-TB may mean that clinicians worldwide will encounter more such cases [5]. It is important to holistically evaluate the risks and benefits while selecting the treatment components for MDR-TB and SLE. Use of low-dose maintenance corticosteroid was necessary for control of SLE in our patient and it did not adversely affect the MDR-TB treatment outcome. Frequent and close monitoring is very important. Pharmacological management should be individualized to achieve optimum disease control with minimum adverse drug events. Developing guidelines for managing MDR-TB in the presence of chronic conditions such as SLE would help clinicians and patients worldwide.

## Consent

Written informed consent was obtained from the patient for this case report and any accompanying images. The consent is available for review by the Editors of the journal.
